# Antiphase synchrony increases perceived entitativity and uniqueness: A joint hand-clapping task

**DOI:** 10.3389/fpsyg.2023.1069660

**Published:** 2023-03-13

**Authors:** Ken Fujiwara, Kunihiko Nomura, Miki Eto

**Affiliations:** ^1^Department of Psychology, National Chung Cheng University, Minxiong, Taiwan; ^2^Faculty of Information Technology and Social Sciences, Osaka University of Economics, Osaka, Japan; ^3^Faculty of Human Sciences, Osaka University of Economics, Osaka, Japan

**Keywords:** synchrony, relative phase, antiphase, in-phase, entitativity, uniqueness, hand-clapping

## Abstract

In- and antiphase are the dominant patterns identified in the study of synchrony in relative phases. Many previous studies have focused on in-phase synchrony and compared it to asynchrony, but antiphase synchrony has yet not been the subject of much research attention. The limited findings on antiphase synchrony suggest that its role or nature is unclear or unstable in human interaction. To account for this factor, this study examined the possibility that antiphase synchrony simultaneously induced perceived entitativity and uniqueness. The results of an experiment employing a joint hand-clapping task supported this prediction. Further, the elevated feeling of uniqueness in those who experienced antiphase synchrony may have increased the self-other overlap for those who felt oneness with their partner, but it decreased overlap for those who did not. The theoretical implications for synchrony literature are discussed.

## 1. Introduction

Interpersonal coordination is defined as “the degree to which the behaviors in an interaction are nonrandom, patterned, or synchronized in both timing and form” (Bernieri and Rosenthal, 1991, p. 403). Previous studies have illustrated that coordinated movement between interactants facilitates perceived entitativity (Lakens and Stel, 2011), rapport (Tickle-Degnen and Rosenthal, 1990; Vacharkulksemsuk and Fredrickson, 2012; Fujiwara et al., 2020), self–other overlap (Lumsden et al., 2014), affiliation (Hove and Risen, 2009), and cooperation behavior (Wiltermuth and Heath, 2009; Cirelli et al., 2014). Although there are substantial differences in the conception and terminology of coordination across studies (see also Burgoon et al., 2014), studies using meta-analysis have also affirmed the robust influence of coordination on positive social outcomes (Rennung and Göritz, 2016; Vicaria and Dickens, 2016; Mogan et al., 2017).

**Figure 1:**
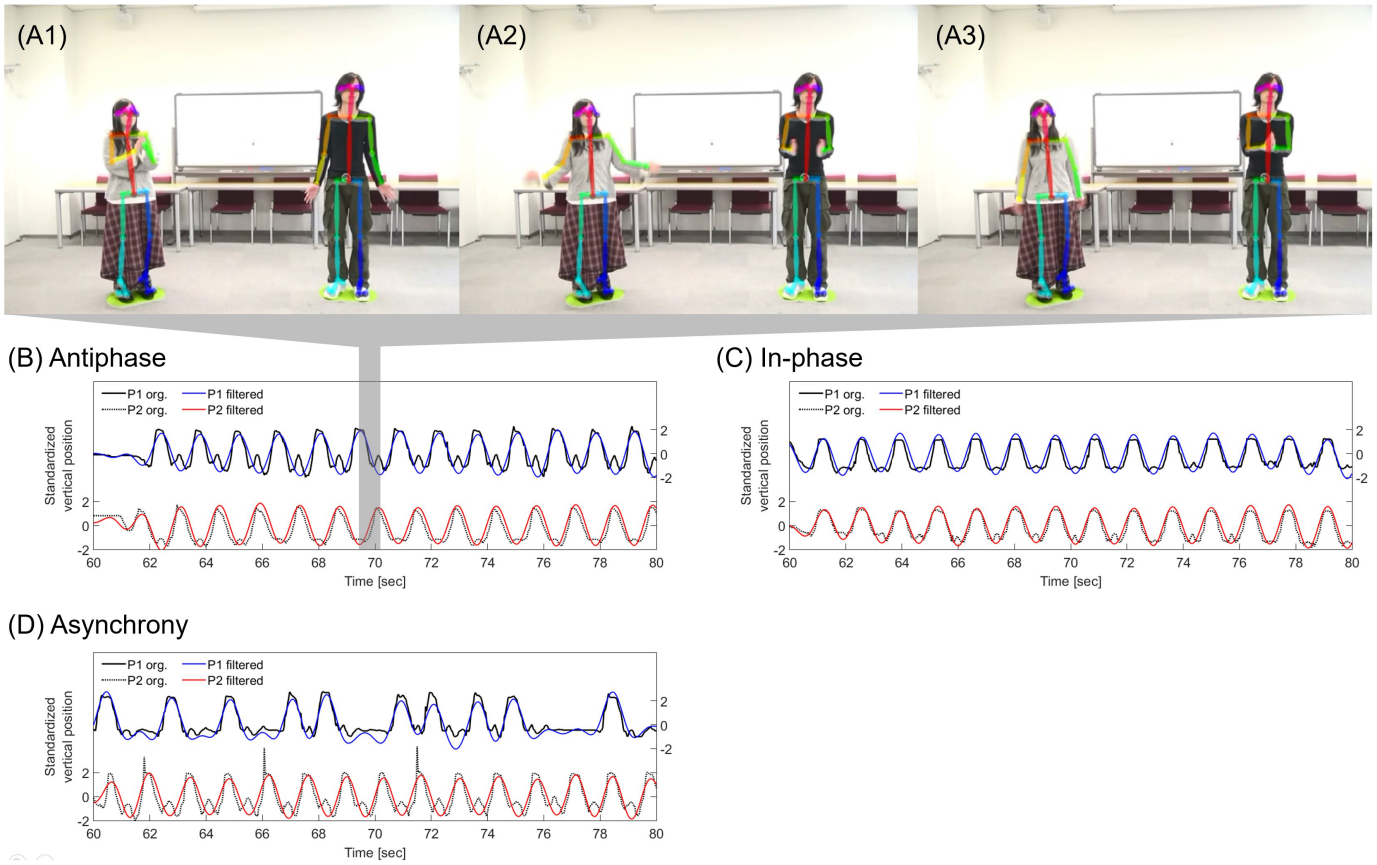
Experimental setting where participants stood on green rubber sheets. **(A1–A3)** represent the sequence in the pair for the antiphase condition. The stick figure overlaid on the participant was generated by OpenPose. **(B–D)** is an example of the two time series in the pair for the antiphase, in-phase, and asynchrony condition, respectively. Each band-pass filtered (0.25–2.00 Hz) time series was represented by red and blue lines on the raw signals.

Bernieri and Rosenthal (1991) identify two major facets in coordination, namely, behavior matching and interactional synchrony. The former identifies similarity in body postures between individuals, whereas the latter reflects active and involved types of coordination, which is further subdivided into interaction rhythms and simultaneous movement (Fujiwara and Daibo, 2022). For instance, according to Schmidt et al. (2014), synchrony is captured according to its degree and its pattern. Interaction rhythms, or the degree of synchrony, indicate rhythmic convergence or temporal coordination, quantified as a coherence measure for spectrum analysis. This measure is used to capture synchrony in a structured or unstructured dyadic conversation (Schmidt et al., 2014; Fujiwara and Daibo, 2016; Fujiwara et al., 2020, 2021; Fujiwara and Yokomitsu, 2021). Alternatively, simultaneous movement, or the pattern of synchrony, is represented as a relative phase measure—as in-phase or antiphase synchrony. In-phase synchrony indicates movements that occur in the same part of the cycle at a given time and is scored with a correlation coefficient of +1 in cross-correlation analysis (Ramseyer and Tschacher, 2011; Kleinbub and Ramseyer, 2021). By contrast, antiphase synchrony refers to movements in opposite parts of their cycles at a given time. In cross-correlation analysis, this pattern is scored using a correlation coefficient of −1 (see also Figure 1). Previous studies on coordination dynamics have indicated that synchronized movement tends to be stable in either in-phase or antiphase patterning at equilibrium points in a coupled-oscillator system (Haken et al., 1985; van Ulzen et al., 2008).

Although in-phase and antiphase synchrony are supposed to be different physical phenomena, to be precise, both have not always been distinguished in the studies of interactional synchrony. Especially, this has been the case when focusing on natural conversations, including therapeutic interactions (e.g., Ramseyer and Tschacher, 2011), in which behaviors were not restricted (except for Fujiwara and Daibo, 2018). For instance, if such studies employ cross-correlation to evaluate synchrony, the absolute value of the cross-correlation coefficient is used as a measure, which disregards whether the temporal coordination observed is in in-phase or antiphase. Still, this could be reasonable because the role of the relative phase becomes less pronounced in individuals who are engaged in unrestricted interactions (Fujiwara and Yokomitsu, 2021; Fujiwara and Daibo, 2022). Also, a practical reason why the distinction between in-phase and antiphase synchrony has been overlooked is that other major synchrony measures that are supposed to assess temporal coordination, such as cross-recurrence quantification analysis (CRQA; Coco and Dale, 2014) and dynamic time warping (Fujiwara et al., 2022a,b), do not account for phase difference. Indeed, several measures are introduced to the research field (Novotny and Bente, 2022), and different measures have little convergent validity (Altmann et al., 2022), which makes it difficult to see the essence of the problem. Even when cross-correlation was computed, some recent studies have shed light on non-absolute values (Nyman-Salonen et al., 2021; Tourunen et al., 2022), which is promising to be an advantage in understanding antiphase synchrony. In this regard, however, perhaps more important in theory is the possibility that antiphase phase synchrony can be regarded as *lagged* in-phase synchrony. In fact, when interactants exhibit antiphase synchrony, their movements will be in-phase synchrony if the timing of one’s movement was shifted by half of the movement cycle. Although to what extent time differences are acceptable as (in-phase) synchrony is still under debate (see also Tschacher et al., 2018), at least no functional difference between in-phase and antiphase synchrony will be found if antiphase synchrony was just lagged in-phase synchrony. And if this is the case, there would be no need to make a strict distinction between them. Indeed, the current study challenges that assumption.

Many previous studies have demonstrated that in-phase synchrony consistently leads to positive social consequences, except for Paulick et al. (2018) that suggests an inverse U-shaped curvilinear relationship. For instance, perceived entitativity is positively associated with in-phase synchrony (Lakens, 2010; Lakens and Stel, 2011). Entitativity refers to the degree to which individuals are perceived as a social unit (Hamilton and Sherman, 1996), which has been verified in the group context (Reddish et al., 2016) but also applied to dyadic interactions (Lakens, 2010). Another major interpersonal outcome is self-overlap which is referred to as the extent of self-rated overlap between oneself and others. The concept is usually measured using a graphical scale, the Inclusion of the Other in Self (IOS) scale (Aron et al., 1992), in which the diagrams consist of increasingly overlapping circles; one representing the self and one representing the other. Self-other overlap seems to be closely linked to rapport (Tickle-Degnen and Rosenthal, 1990), perceived similarity and mutual liking (Valdesolo and DeSteno, 2011; Rabinowitch and Knafo-Noam, 2015), connectedness (Valdesolo et al., 2010), and affiliation (Hove and Risen, 2009). In relation to synchrony, Lumsden et al. (2014) demonstrated that the interactants perceived a greater self-other overlap with their partner following a period of synchronous movement. Entitativity and self-other overlap should share some underlying assumptions, however, they are not the same (Lakens and Stel, 2011; Edelman and Harring, 2015). The latter tends to lay emphasis on positive affective relations between the interactants, while the former is nuanced to functionality as a unit and does not necessarily imply that the interactants are emotionally connected. In other words, entitativity does not require the interactants to overlap as long as they function as a unit. Although the goal of the current study is not to draw a causal relationship between entitativity and self-other overlap (see also Lakens and Stel, 2011), this study targets both since the contrast between them is expected to contribute to a better understanding of the role of temporal coordination.

Although the association between synchrony and prosocial consequences appears robust, research on antiphase synchrony yielded mixed evidence. For instance, individuals perceived the same level of rapport for the movement of in- and antiphase synchrony in one study (Miles et al., 2009); however, in another, antiphase synchrony led to a lower level of prosociality, at a similar level to that of asynchrony (Cross et al., 2016). Furthermore, individuals in antiphase synchrony showed a greater physiological change (i.e., endorphin release), implicating social bonding (Dunbar and Shultz, 2010), than others in in-phase conditions (Sullivan and Blacker, 2017).

Why is the social role of antiphase unclear or unstable? Theoretical frameworks based in the motor system and neural computation do not seem to be able to provide a sufficient explanation for this. The Haken–Kelso–Bunz model (Haken et al., 1985), a leading model based on the dynamic systems approach, posits that in-phase and antiphase coordination patterns are similarly dominant and stable unless or until they exceed a critical frequency threshold or a physical bifurcation point (≈1.4 Hz, Schmidt et al., 1990). In commonly used rhythmic tasks, such as gait (van Ulzen et al., 2008), drumming (Sullivan and Blacker, 2017), rowing (Sullivan et al., 2014), and moving joystick (Cross et al., 2016), rhythm does not reach an excessive speed, being limited to about 1 Hz (60 beats per minute), to allow antiphase synchrony to be stable. On this interpretation, antiphase synchrony does not lead to socially favorable outcomes is not rooted in the difficulty of achieving a given pattern of synchrony. On this view, a neural computation model would not add any important perspective. According to the general principle of optimization, such as the reduction of prediction errors (Koban et al., 2019), a state that is high in predictability is considered highly favorable. Given that in- and antiphase synchrony are equally stable, the brains taking part in the interaction are considered to mutually predict each other and therefore to be equally preferable. Since these models do not distinguish between in- and antiphase synchrony in terms of stability or favorableness, these alone cannot account for the instability of the relationship between antiphase synchrony and prosocial outcomes.

Rather, the unclarity or instability of antiphase may be attributed to its mixed property, in which the movement in the same rhythm and with different (opposing) timings are combined. Antiphase synchrony can facilitate perceived oneness (or entitativity, Lakens and Stel, 2011) because the movement in the antiphase synchrony keeps the same rhythm with the partner, a preferable state between interactants in terms of predictability (Koban et al., 2019). However, antiphase synchrony may not lead to a predicted prosocial consequence if attention is directed to differences between interlocutors. In that synchrony facilitates perceived similarity (Valdesolo and DeSteno, 2011), it may seem difficult to focus on differences. However, recent studies have shown that interactants even in in-phase synchrony did not lose their individual identity (Good et al., 2017) and maintained a high level of self-control (i.e., agency) over their own movement while contributing equally to joint action (Reddish et al., 2020). The case of antiphase synchrony will become more salient as it requires more attention between partners than in-phase synchrony (Sullivan and Blacker, 2017). Indeed, Miles et al. (2010) found that antiphase synchrony enhanced the attention to oneself. Thus, presumably due to performing movements with the opposite timing to one’s partner, individuals in antiphase synchronous movement pay attention to themselves and the difference from their interactant, which results in the salience of self-identity. In this study, the perception of self-identity was measured as the sense of uniqueness, i.e., the sense of being distinct from others (Lynn and Harris, 1997; Şimşek and Yalınçetin, 2010).

Given this, we predicted that interactants in antiphase synchrony could experience entitativity and uniqueness at the same moment. To this end, we tested the following hypotheses:

*H1*: Participants in the antiphase synchrony condition will perceive entitativity **(a)** as much as those in the in-phase synchrony condition but **(b)** more than those in the asynchrony condition.

*H2*: Participants in the antiphase synchrony condition will perceive uniqueness **(a)** as much as those in the asynchrony condition but **(b)** more than those in the in-phase synchrony condition.

*H3*: Perceived entitativity and uniqueness will be positively and significantly correlated in the antiphase synchrony condition.

Further, we also measure self-other overlap (Aron et al., 1992). Because of the inconsistent findings of the previous studies on antiphase synchrony and emotional connection between interactants (Miles et al., 2009; Cross et al., 2016; Sullivan and Blacker, 2017), this study did not hypothesize about self-other overlap. Rather, the extent of self-other overlap in each condition and its relationship to perceived entitativity and uniqueness is explored for the purpose of better understanding the role of antiphase synchrony.

## 2. Methods

### 2.1. Participants

To select a sample size, a previous study with a similar design (i.e., a single between factor with three levels; antiphase, in-phase, asynchrony) was reviewed. Since Macrae et al. (2008) had reported an *F* value [*F* (2, 27) = 10.50], following Lakens (2013), we calculated the effect size ($\eta_{p}^{2}$ = 0.4375). Then, the necessary sample size was estimated using the *pwr.anova.test* function in the “pwr” package in R, which showed the sample size of 7 is necessary for each group to achieve the power 0.90 in the level of significance of 0.05. While a sample of 30 in total (10 in each condition) was considered powerful enough to investigate the effect of synchrony on attention tendencies between synchronous interactants, more than twice as many participants were recruited to reduce Type II errors. We recruited 70 undergraduates from a Japanese university, and 67 participated in the experiment in exchange for extra course credit. The participants were randomly assigned to in-phase, antiphase, and asynchrony conditions of the between-subject design. However, one participant was removed from the analysis due to incorrect implementation of hand-clapping. In all, 66 undergraduates (27 females, 39 males, *M*_age_ = 20.56, SD_age_ = 3.46) made up the subject of analysis.

### 2.2. Hand-clapping task

The participants were grouped into pairs with a confederate (Figure 1). First, the participants read and signed an informed consent, then they were instructed to engage in a joint hand-clapping task, which was made up of three sessions: control, experimental, and additional sessions. As each session consisted of 3 min and 20 s, the total length of the experiment was about 20 min. The hand-clapping task was video recorded for the subsequent synchrony analysis.

In the control (first) session, the participants were asked to clap their hands together with their partner (i.e., confederate) to the rhythm of a metronome with a tempo of 0.73 Hz (44 beats per minute). They were also required to clap their hands in front of their chest and lower their hands to waist level in the middle of the clapping so that the hand movements could be represented as the vertical position of the hands. We did not specify the extension or flexion of the elbows. After 20 s of clapping, the participants took a 10-s rest, and this pattern was repeated seven times during the session. Following the session, each participant completed a questionnaire on perceived entitativity, uniqueness, and self-other overlap.

In the experimental (second) session, the participants were again asked to clap along with the metronome, as in the control session. Their partner was instructed to clap with the same emphasis on the vertical motion, but to be free with the timing. The partners then clapped according to the condition assigned before the experiment; same timing (in-phase), opposite timing (antiphase), and at random (asynchrony). The metronome tempo and timing during this session were identical to that of the first session. Following the session, each participant completed a questionnaire on perceived entitativity, uniqueness, and self-other overlap. This session was the focus of the current study, and in order to provide a clear contrast with the first session, the change in scores from the first session to the second session was used when analyzing the self-reported measurements.

In an additional (third) session, the participants were instructed to clap freely and their partner was asked to clap along with the metronome as in the control session. Because the experimental manipulation was not assigned, the data obtained in this session are not reported here, but they are given in the Supplementary material (Supplementary Table S1).

### 2.3. Questionnaires

Perceived entitativity was measured using four items from Lakens (2010) on a 7-point scale ranging from 1 (not at all) to 7 (very much), *α* = 0.831–0.923. As a measure of self-other overlap, a single graphical item of the IOS scale (Aron et al., 1992) was used. The participants were asked to choose a pair of circles from seven with different degrees of overlap (1 = no overlap, 2 = little overlap, 3 = some overlap, 4 = equal overlap, 5 = strong overlap, 6 = very strong overlap, 7 = most overlap). Regarding perceived uniqueness, four items were created for this study with reference to Lynn and Harris (1997) as well as Şimşek and Yalınçetin (2010): “Both people were autonomous,” “Each person showed his or her own individuality,” “Each person demonstrated his or her own uniqueness,” and “Each person actively expressed his or her own presence.” It was a 7-point scale ranging from 1 (not at all) to 7 (very much), *α* = 0.773–0.836.

### 2.4. Quantifying phase synchrony

To ensure that the participant and the confederate exhibited stable movement patterns with appropriate timing according to the experimental conditions, the phase synchrony of their hand movements was calculated. In this study, the participant’s and confederate’s hand movements during the task were captured using 2D pose estimation software, OpenPose 1.5.1 (Cao et al., 2019). This technique, which incorporates computer vision and deep learning, automatically detects the 2D coordinates of the joint parts in the human body from a video clip. Since the participants were instructed to clap their hands with an emphasis on vertical motion, the hands clapped when the vertical position of the hands is at the top, which is represented by the waveform in Figure 1; when it is at the bottom, the hands are at waist level, not being clapped.

**Figure 2:**
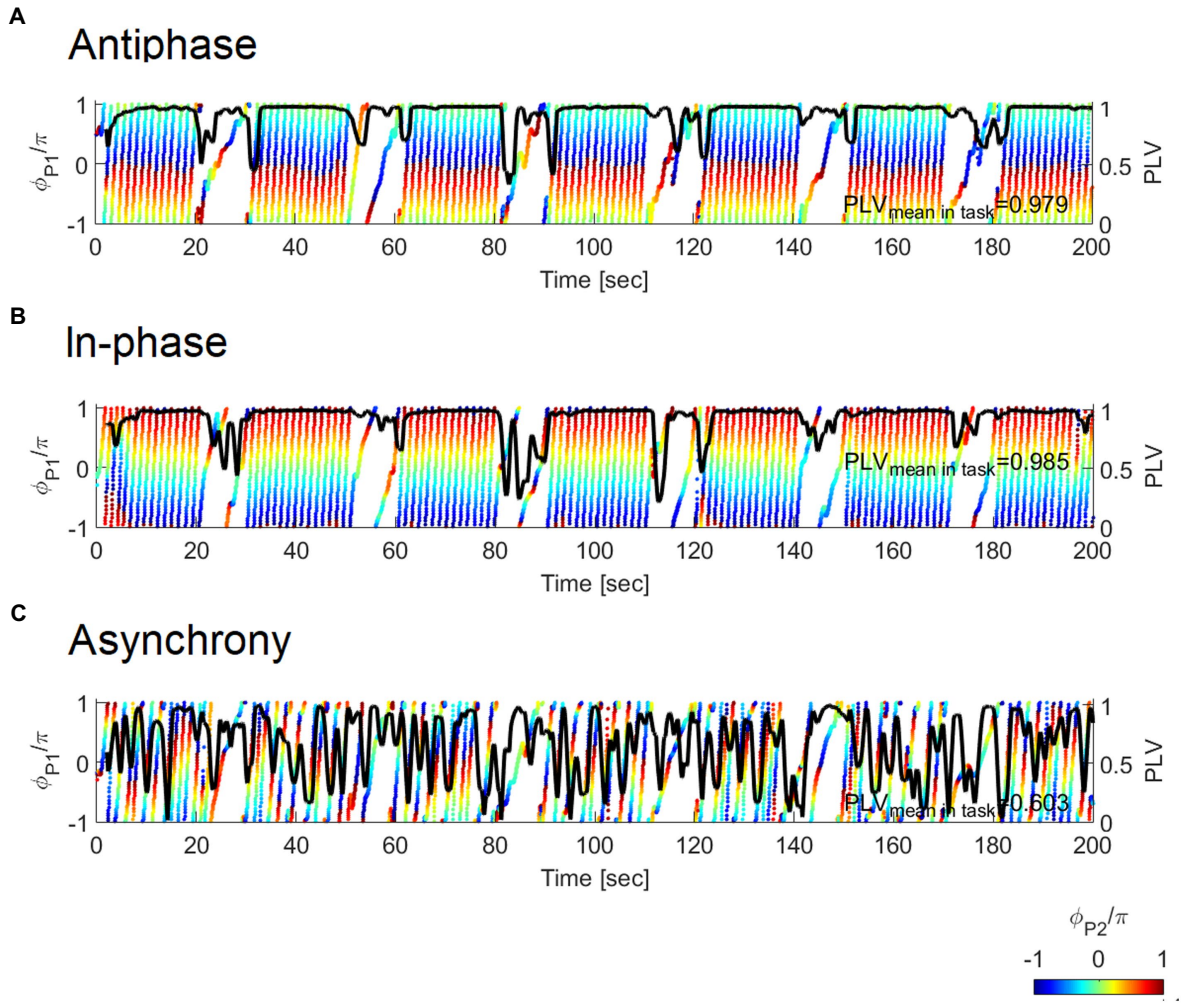
Example of the relative phase and PLV between the handclap motions of the participant (P1) and confederate (P2) in the **(A)** Antiphase, **(B)** In-phase, and **(C)** Asynchrony conditions. The color synchrogram illustrates a relative phase between the two types of participants. Colored dots represent the instantaneous phase of P1($\phi_{P1}$ ). The diagonal arrays of dots in the plot demonstrate the increase of $\phi_{P1}$ from −π to π. The color on dots shows the instantaneous phase of P2($\phi_{P2}$ ) as $\phi_{P1}$ gains 2π from −π to π. Here −π, 0, and π are displayed in blue, green, and red, respectively. Phase synchronization is represented by horizontal lines of the same color band. PLV, indicated by the thick black line, is stable with a high value across trials in both the antiphase and in-phase synchrony conditions but unstable in the asynchrony condition.

Using the vertical position of the right hand of the participant and the confederate, we performed synchrony analysis (Doesburg et al., 2008) to quantify the phase synchrony. First, we applied spline interpolation to the coordinate time series to address missing values, then applied a 10th-order Butterworth band-pass filter with a lower cutoff frequency of 0.25 Hz and a higher cutoff frequency of 2.0 Hz (Figure 1). With the time series obtained, we performed the Hilbert transform in the MATLAB Signal Processing Toolbox to calculate the analytic signal, then calculated phase-locking value (PLV), the most commonly used measure of the phase synchrony (Lachaux et al., 1999), to ensure the phase synchrony was achieved in in-phase and antiphase synchrony condition, compared to the asynchrony condition. The synchronization of periodic self-oscillatory systems is often-defined as phase locking:


$$\left|n\phi_{1}-m\phi_{2}\right|<\mathrm{constant}\text{,}$$

where *n* and *m* are integer numbers. In this study, *n = m = 1* because the experimental conditions of in-phase and antiphase handclap produce the same rhythm for both types of participants. We then calculated a phase difference between paired data, and based on that, a PLV was computed. Following the previous studies (Lachaux et al., 1999; Nomura et al., 2003; Richardson et al., 2012), we define the PLV at time *t* as the average value:


$$\mathrm{PL}V_{t_{k}}=N^{-1}|\overset{k}{\underset{n=k-N}{\sum }}e^{i|\phi_{1}\left(t_{n}\right)-\phi_{2}\left(t_{n}\right)|}|$$

where *t_k_* is time of the *k*-th sampling point, and *N* is the time window that works as in the case of cross-correlation. In this study, we set *N* = 60 to represent a time window of 2 s. PLV is a real value ranging from 0 to 1 and is close to 1 where the phase difference varies little across the trails; namely, the phase synchrony was achieved (Figure 2). The mean PLV value during 20 s of clapping over 7 repetitions was used in the subsequent analysis.

**Figure 3:**
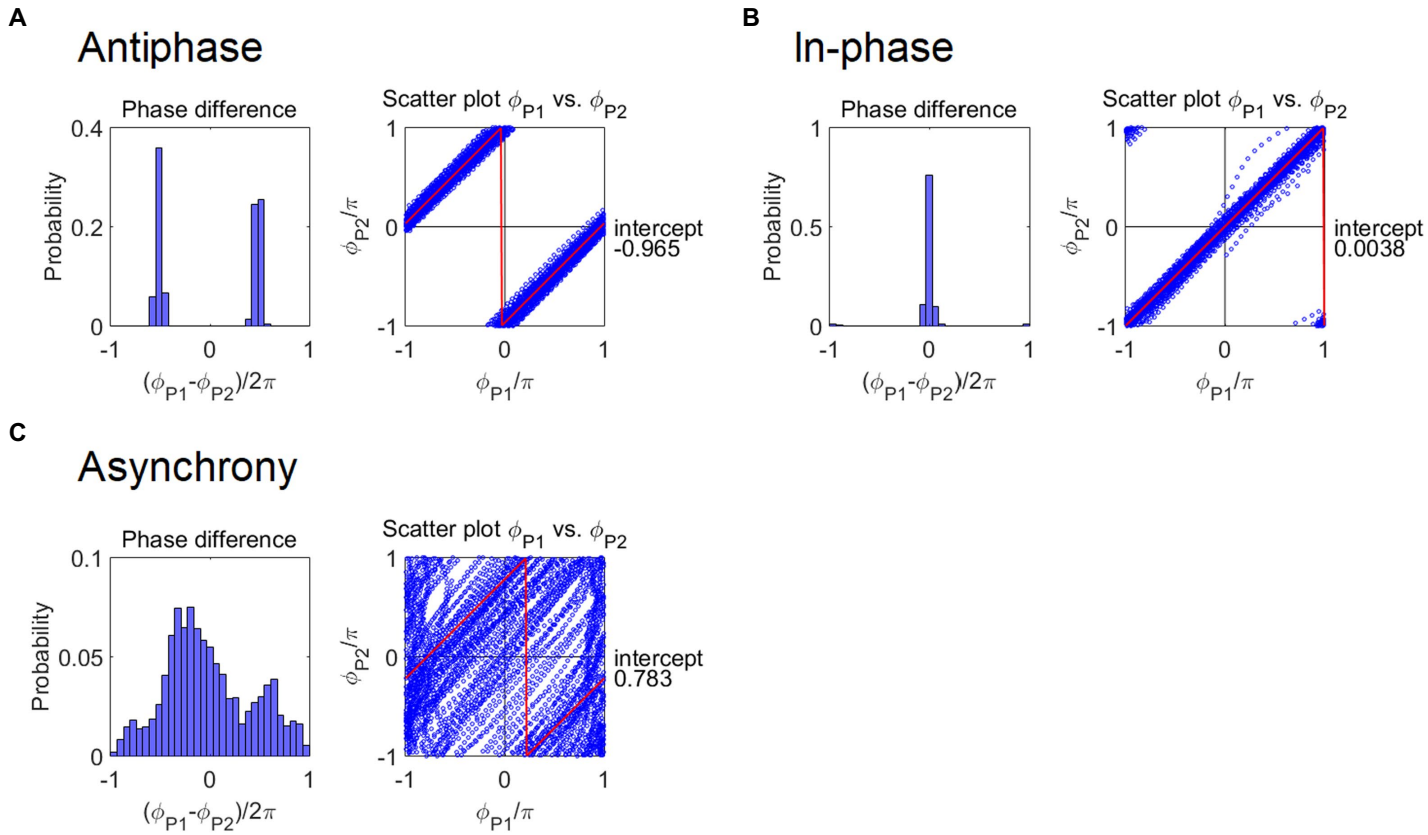
Example of a distribution (Left) and a scatter plot (Right) of the phase difference between the handclap motions of the participant (P1) and confederate (P2) in **(A)** Antiphase, **(B)** In-phase, and **(C)** Asynchrony conditions. The distribution of the antiphase condition presented a bimodal peak around −π and π, whereas that in the in-phase condition showed a sharp peak around zero. In the asynchrony condition, the distribution shape was unspecified. In the scatter plot of the phase difference, red lines represent the regression lines fitted with the sawtooth wave function $\left[f\left(x\right)\right]\text{.}$The intercept in the plot, *f*(0), represents the phase difference.

Although PLV indicates the degree of phase synchrony, it does not distinguish between in-phase and antiphase patterns. To compute the value of phase difference, we also present the data in a scatter plot between $\phi_{1}$ and $\phi_{2}$ (Figure 3). Then, we used a fitted line plot to display a sawtooth wave with amplitude π and period [−π, π] in the following equation


fx=2x+1−a2−x+1−a2+12

where ⌊⋅⌋ represents the floor function, and a is the displacement along the horizontal axis. For example, if variable a is substituted for −π or π, this sawtooth wave corresponds a diagonal line in the case of a range from −π to π in both axes. The intercept coefficient of the fitting line as a phase difference was then obtained (Figure 3). If participants complete an antiphase handclap, the intercept coefficient is close to π or − π, and it is close to 0 in the in-phase handclap. The absolute value of the intercept coefficient was adopted in the statistical testing. Root mean square (RMS) was also calculated to assess a variation from the regression line, which indicates the degree of phase synchrony as with the PLV whereas it was obtained through a different computational process from that of the PLV.

## 3. Results

### 3.1. Manipulation check: Phase synchrony

For the manipulation check, the PLV, the intercept coefficient of the sawtooth wave, and the RMS was submitted to a three (synchrony: antiphase, in-phase, asynchrony) by two (session: first, second) mixed ANOVA, using synchrony and session as the between–and within-subject factors, respectively (Table 1). For the PLV, the two-way interaction was significant [*F* (2,63) = 199.67, *p* < 0.001, *η*^2^ = 0.333]. The simple effect of synchrony in the first session was not significant [*F* (2,63) = 0.93, *p* = 0.399, *η*^2^ = 0.029], whereas this effect was significant in the second session [*F* (2,63) = 226.90, *p* < 0.001, *η*^2^ = 0.878]; Holm’s multiple comparison revealed that the difference was not significant between the antiphase and in-phase conditions [*t* (63) = 0.55, adjusted *p* = 0.582], whereas the difference between antiphase and asynchrony conditions [*t* (63) = 17.94, adjusted *p* < 0.001] and in-phase and asynchrony conditions [*t* (63) = 18.91, adjusted *p* < 0.001] was significant. These results showed that the phase synchrony was successfully achieved and the phase difference was stable across trials in the in-phase and antiphase synchrony conditions compared to asynchrony condition.

**Table 1:** Phase synchrony of the first and second session in each condition.

Condition	*n*	PLV				Intercept coefficient				RMS			
		1st		2nd		1st		2nd		1st		2nd	
		*M*	SD	*M*	SD	*M*	SD	*M*	SD	*M*	SD	*M*	SD
Antiphase	21	0.976	0.017	0.963	0.019	0.063	0.091	0.926	0.036	0.346	0.033	0.362	0.043
In-phase	23	0.978	0.010	0.971	0.016	0.041	0.047	0.047	0.079	0.332	0.035	0.338	0.075
Asynchrony	22	0.972	0.020	0.680	0.086	0.050	0.045	0.665	0.277	0.349	0.047	0.766	0.025

PLV, Phase-Locking Value; RMS, Root Mean Square; 1st, Session 1 (Control); 2nd, Session 2 (Experimental).

Likewise, for the intercept coefficient, the two-way interaction was significant [*F* (2,63) = 157.62, *p* < 0.001, *η*^2^ = 0.226]. The simple effects of synchrony in the first session were not significant [*F* (2,63) = 0.64, *p* = 0.530, *η*^2^ = 0.020], whereas those in the second session were significant [*F* (2,63) = 161.24, *p* < 0.001, *η*^2^ = 0.837]; Holm’s multiple comparison indicated that the difference was significant between antiphase and asynchrony conditions [*t* (63) = 5.10, adjusted *p* < 0.001], the in-phase and asynchrony conditions [*t* (63) = 12.38, adjusted *p* < 0.001], and the antiphase and in-phase conditions [*t* (63) = 17.37, adjusted *p* < 0.001]. Thus, the confederate and participant in the antiphase conditions moved in the opposite directions while those in in-phase condition moved synchronously.

For the RMS, two-way interaction was also significant [*F* (2,63) = 314.18, *p* < 0.001, *η*^2^ = 0.343]. The simple effect of synchrony in the first session was not significant [*F* (2,63) = 1.28, *p* = 0.285, *η*^2^ = 0.039], while that in the second session was significant [*F* (2,63) = 460.80, *p* < 0.001, *η*^2^ = 0.936]; Holm’s multiple comparison revealed that the difference was not significant between antiphase and in-phase conditions [*t* (63) = 1.52, adjusted *p* = 0.133], whereas the difference between antiphase and asynchrony conditions [*t* (63) = 25.16, adjusted *p* < 0.001] and in-phase and asynchrony conditions [*t* (63) = 27.28, adjusted *p* < 0.001] was significant. This means that the phase difference of asynchrony conditions did not show a clear pattern, compared to the in-phase and antiphase synchrony conditions.

### 3.2. Impact of phase synchrony on perceived entitativity, uniqueness, and self-other overlap

Descriptive statistics are shown in Table 2. For the primary test, the change in score of the perceived entitativity, uniqueness, and IOS (self-other overlap) from the first to the second session was submitted to a between-factors ANOVA (synchrony: antiphase, in-phase, asynchrony). For entitativity, we predicted that participants in the antiphase synchrony condition would perceive entitativity equivalently with those in the in-phase synchrony condition (H1a), but more than those in the asynchrony condition (H1b). A significant difference was found among the three conditions [*F* (2, 63) = 18.02, *p* < 0.001, *η*^2^ = 0.364]. Subsequent Holm’s multiple comparisons indicated that the difference between antiphase and in-phase conditions was not significant [*t* (63) = 1.05, adjusted *p* = 0.298], while the differences between the antiphase and asynchrony conditions [*t* (63) = 5.60, adjusted *p* < 0.001] and in-phase and asynchrony conditions [*t* (63) = 4.67, adjusted *p* < 0.001] was significant.

**Table 2:** Impact of phase synchrony on perceived entitativity, uniqueness, and IOS (self-other overlap).

Condition	*n*		Entitativity			Uniqueness			IOS		
			1st	2nd	Change	1st	2nd	Change	1st	2nd	Change
Antiphase	21	*M*	3.595	3.452	−0.143^a^	3.619	4.274	0.655^a^	3.714	3.571	−0.143^a^
		SD	1.059	1.201	1.122	1.239	1.447	1.345	1.793	1.748	2.104
In-phase	23	*M*	4.489	3.989	−0.500^a^	3.609	3.326	−0.283^b^	4.174	3.609	−0.565^ab^
		SD	1.046	1.354	1.003	1.457	1.086	1.295	1.850	2.017	1.308
Asynchrony	22	*M*	4.159	2.091	−2.068^b^	4.045	5.045	1.000^a^	3.727	2.136	−1.591^b^
		SD	1.068	0.981	1.247	0.878	1.112	0.957	1.723	1.082	2.130

The different superscripts (a, b) in the Change column represent significant differences in the change in score between Antiphase, In-phase, and Asynchrony conditions on Holm's multiple comparisons (adjusted *p* < 0.05) with the one-way ANOVA. 1st, Session 1 (Control); 2nd, Session 2 (Experimental).

We hypothesized that participants in the antiphase synchrony condition would perceive uniqueness equivalently with those in the asynchrony condition (H2a) but to a greater degree than those in the in-phase synchrony condition (H2b). As with entitativity, the result indicated that the difference among three conditions was significant [*F* (2, 63) = 6.78, *p* = 0.002, *η*^2^ = 0.177]. Subsequent Holm’s multiple comparisons revealed that the difference between asynchrony and antiphase conditions was not significant [*t* (63) = 0.93, adjusted *p* = 0.354], whereas the difference between asynchrony and in-phase conditions [*t* (63) = 3.55, adjusted *p* = 0.002] and antiphase and in-phase conditions [*t* (63) = 2.57, adjusted *p* = 0.025] was significant.

For IOS, which measures of self-other overlap, a significant difference was seen among three conditions [*F* (2, 63) = 3.42, *p* = 0.039, *η*^2^ = 0.098]. Subsequent Holm’s multiple comparisons revealed that the difference between antiphase and asynchrony conditions was significant [*t* (63) = 2.53, adjusted *p* = 0.042], whereas the difference between antiphase and in-phase conditions [*t* (63) = 0.75, adjusted *p* = 0.458] and in-phase and asynchrony conditions [*t* (63) = 1.83, adjusted *p* = 0.143] was not significant.

### 3.3. Correlation of entitativity, uniqueness, and IOS

The values for Pearson’s correlation between entitativity, uniqueness, and IOS were calculated in each condition (Table 3). In the first session, entitativity was not significantly correlated with uniqueness in all conditions. On the contrary, as predicted in H3, the correlation was significant in the antiphase condition in the second session (*r* = 0.650, *p* < 0.001) but not in the in-phase and asynchrony conditions. The IOS was significantly correlated with entitativity throughout the entire condition, except for the asynchrony condition in the second session. However, no significant correlation was seen between the IOS and uniqueness, while its coefficient was not small in the asynchrony condition (*r* = −0.416 in the first session and *r* = −0.401 in the second session).

**Table 3:** Correlations and partial correlations between entitativity, uniqueness, and IOS (self-other overlap).

	Antiphase				In-phase				Asynchrony		
Session1											
	Ent.	Uniq.	IOS		Ent.	Uniq.	IOS		Ent.	Uniq.	IOS
Ent.	-	0.152	0.576^**^	Ent.	-	−0.020	0.700^**^	Ent.	-	0.094	0.520^*^
Uniq.	0.143	-	−0.061	Uniq.	0.005	-	0.032	Uniq.	−0.148	-	−0.402
IOS	0.575^**^	0.033	-	IOS	0.700^**^	0.026	-	IOS	0.529^*^	−0.416	-
Session 2											
	Ent.	Uniq.	IOS		Ent.	Uniq.	IOS		Ent.	Uniq.	IOS
Ent.	-	0.660^**^	0.712^**^	Ent.	-	0.331	0.842^**^	Ent.	-	−0.093	0.235
Uniq.	0.650^**^	-	−0.319	Uniq.	0.235	-	−0.248	Uniq.	−0.198	-	−0.366
IOS	0.704^**^	0.286	-	IOS	0.831^**^	0.061	-	IOS	0.291	−0.401	-

Ent., Entitativity; Uniq., Uniqueness. Values below the diagonal line indicate Pearson’s bivariate correlation, and those above the diagonal line show the partial correlation between the two variables controlling for the other variable. Session 1: Control session. Session 2: Experimental session. *p < 0.05, **p < 0.01.

With respect to the second session of antiphase condition, which showed a characteristic positive association between entitativity and uniqueness, an additional finding was identified; the correlation between uniqueness and IOS was reversed if entitativity was controlled (from *r* = 0.286 to *r*_partial_ = −0.319, ∆*r* = 0.605). No such inversion or large difference value was seen from controlling the impact of entitativity in any other condition (∆*r*s < 0.309). Likewise, no impact was seen on the correlation between entitativity and uniqueness (/IOS) when IOS (/uniqueness) was controlled.

## 4. Discussion

### 4.1. Stability of the phase difference during hand-clapping task

A manipulation check found that our confederate successfully generated a phase difference following the experimental condition. The result of PLV indicated that in comparison to the asynchrony condition, phase difference was stable across trials in the in-phase and the antiphase synchrony conditions. Previous studies have found that synchronized movement could be stable in either the in-phase or antiphase patterning (Haken et al., 1985; van Ulzen et al., 2008). Because the tempo of the hand-clapping task in this study was slower than the physical bifurcation point (≈1.4 Hz, Schmidt et al., 1990), a confederate could as easily keep moving in in-phase and antiphase patterning. The intercept coefficient of the regression line in a scatter plot showed that the phase difference of the first session and in-phase condition in the second session was close to 0, indicating that the confederate and the participant moved synchronously at the same timing. On the contrary, the phase difference of the antiphase condition was close to 1 (i.e., π or − π), which shows that the two were moving in the opposite timing. RMS showed that the relative phase between the participant and the confederate in in-phase and antiphase condition exhibited a similarly subtle variance. However, the phase difference of the asynchrony condition was unstable across trials and did not show a clear pattern, which supports the PLV results with a different (regression-based) approach. All in all, our manipulation in-phase and antiphase synchrony functioned as intended.

### 4.2. Role of antiphase synchrony

This study focused on the social role of antiphase synchrony. In particular, the possibility that interactants in antiphase synchrony simultaneously experience entitativity and uniqueness was examined. The results supported our prediction; that is, the participants in the antiphase synchrony condition perceived a similar entitativity (change from the first session) to that from the in-phase synchrony condition (H1a), at a significantly higher rate than those in the asynchrony condition (H1b). The antiphase synchrony generated a sense of uniqueness in a similar way to that of the asynchrony condition (H2a) and significantly more than in the in-phase condition (H2b). Further, more directly, the perceived entitativity and uniqueness were significantly correlated only when the participants clapped their hands at opposite timing (i.e., the antiphase condition at the second session), which supported H3. These findings constitute the first evidence demonstrating that the interactants in the antiphase synchrony simultaneously felt entitativity and uniqueness. The different functionality from in-phase synchrony suggests that antiphase synchrony appears to be more than just *lagged* in-phase synchrony.

In previous studies, the study of antiphase synchrony has produced mixed evidence, while in-phase synchrony has consistently impacted social variables (Miles et al., 2010; Cirelli et al., 2014; Sullivan et al., 2014; Cross et al., 2016; Sullivan and Blacker, 2017). This cannot be explained using the framework in the motor system and neural computing (Haken et al., 1985; Koban et al., 2019) as they do not distinguish between in- and antiphase synchrony in terms of system stability and preferability. In this regard, by shedding light on self-identity (i.e., perceived uniqueness), this study could produce an explanation of why the role of antiphase synchrony remains unstable; perceived entitativity and uniqueness are enhanced through the mixed property of antiphase synchrony, in which the movement in the same rhythm and at different (opposite) timings are combined. The interactants may have perceived entitativity because their movements were in the same rhythm, which provided predictability and likability, similar to in-phase synchrony. At the same time, movements with different timing in antiphase synchrony could elevate the level of attention (Sullivan and Blacker, 2017) that is directed at oneself (Miles et al., 2010), which would result in an increase of the perceived uniqueness.

Perceived entitativity and uniqueness can affect interpersonal relationships (i.e., self-other overlap) differently. Our results provided partial support for this notion; perceived entitativity was positively associated with the IOS in many conditions. Perceived uniqueness, however, showed a variant impact on the self-other overlap; its correlation was nearly zero under several conditions, while it showed a negative correlation with sizable coefficients (*r* ≈ −0.40) in the asynchrony condition. More importantly, during the second session in the antiphase condition, perceived uniqueness had a positive association with the self-other overlap, but the correlation was reversed when the entitativity was controlled. This means that in individuals who experienced antiphase synchrony, the feeling of uniqueness increased the self-other overlap when the focus fell on the connection with their partner. However, the elevated sense of uniqueness may have diluted the association with prosocial variables when they participants were not paying close attention to the connection. Indeed, after controlling the effect of entitativity, uniqueness lowered the self-other overlap, as indicated by the partial correlation coefficient between the perceived uniqueness and IOS. This variability may be the reason that antiphase synchrony produces mixed evidence. Although neither simple nor partial correlation coefficients were significant in the sample size of this study (around 20 each across three conditions), the coefficients obtained should not have negligibly small effect sizes (i.e., *r* = 0.286, *r*_partial_ = −0.319 in Table 3). It should also be noted that the difference in coefficient was large (i.e., ∆*r* = 0.605) and straddled zero. In the future, to reach a clearer view of the moderating effect of attention on the association between uniqueness and self-other overlap or other social variable, we must experiment with manipulating the direction of attention (oneself vs. partner or their connection).

The current findings may offer practical implications in our daily interactions as well as more specific situations such as therapeutic interactions. Indeed, recent studies confirmed that antiphase synchrony as well as in-phase synchrony was observed in a therapy situation (Nyman-Salonen et al., 2021; Tourunen et al., 2022). Given that our conversation proceeds through a sequence of turn-taking (Stivers et al., 2009), in-phase synchrony seems to be achieved when a listener moves (e.g., nodding) in response to the speaker’s speaking behavior. As illustrated in the previous studies (Tickle-Degnen and Rosenthal, 1990; Valdesolo et al., 2010; Valdesolo and DeSteno, 2011; Rabinowitch and Knafo-Noam, 2015), it should come as no surprise that such conversations represent the mutual interest and promote the perceived entitativity as well as self-other overlap. Alternately, antiphase synchrony can be thought of as a case where the listener does not move when the speaker is speaking but instead moves in the same way as the speaker when the listener gets a turn. In order to preserve the autonomy of the conversation partner or therapy patient while not compromising its functionality as a unit of interaction, this type of interaction is likely to be more effective.

### 4.3. Limitation and future directions

The experimental design used could be considered to be a limitation of this study. To clearly illustrate the role of the antiphase, this study used a mixed design. At first, the participants clapped their hands in phase, and then they clapped in assigned conditions including in antiphase. This experiment could contribute to clarifying the differences between the in-phase and antiphase synchrony conditions. Indeed, the order of the experimental assignment to contrast the synchronous movement, such as synchrony-asynchrony versus asynchrony-synchrony, could have a substantial impact on the perception of synchrony (Marques-Quinteiro et al., 2019). In future research, experiments with only one session, in which the participants have no anchor, should be conducted. In addition, the role of a metronome may be reconsidered. As in the previous studies (Macrae et al., 2008; Miles et al., 2010), we set a metronome to keep the pace constant (0.73 Hz, 44 beats per minute) so that the confederate make the phase synchrony more salient at the first (control) session and in in-phase and antiphase synchrony condition at the second (experimental) session. However, this means the participant’s movement was synchronized in in-phase with the metronome even in the “antiphase” synchrony condition. Given that the task was introduced as a joint task and the questionnaire emphasized the relation to the partner, the participant’s attention should be directed to their partner, not to the metronome, such that the effect of the metronome is believed to be minimal. Still, an experimental setting without a metronome or any other external signals should be included in future research. Relatedly, note that the primary findings were obtained in conditions of *perceived* synchrony, where the partner seeks to (dis)engage in synchrony (Cacioppo et al., 2014; Koehne et al., 2016). It would appear worthwhile to investigate the *produced* synchrony in which one attempts to achieve synchrony spontaneously. To address this concern, we allowed the participants to clap their hands freely in the third session. However, perhaps due to their experience in the earlier sessions, they showed little synchrony (see Supplementary material). There seems to be a tradeoff, such that asking participants to clap in a specific pattern may undermine their autonomy, but experimental manipulations should be employed to investigate the role of synchrony produced by antiphase interaction. Finally, a boundary condition on the relationship between antiphase synchrony and social outcome should also be imposed. The current findings suggest that whether attention is directed to the connection between the interactants or to oneself can determine whether antiphase synchrony leads to prosocial consequences. In this regard, individual differences, such as attention bias toward oneself, might be a promising moderator.

Another line of future work on antiphase synchrony may be in conjunction with physiological synchrony. Indeed, Tschacher and Meier (2020) identified in-phase as well as antiphase synchrony in the physiological signals (i.e., respiration and heart rate) between a therapist and her clients, which were associated with ratings of the therapy process. Tourunen et al. (2022), focusing on a couple therapy situation, also revealed both types of phase synchrony in electrodermal activity by means of skin conductance, both of which interplayed with movement synchrony in a different way. These studies will untangle the complexity of synchrony phenomena, e.g., whether physiological synchrony and movement synchrony are governed by the same theory. Antiphase synchrony has not received much attention in the literature, but it could provide a theoretical extension to synchrony research if we unravel the principles hidden in its instability.

## Data availability statement

The datasets presented in this study can be found in online repositories. The names of the repository/repositories and accession number(s) can be found at: https://osf.io/6kxne/.

## Ethics statement

All procedures performed in this study were approved by the Ethical Committee of the Faculty of Information Technology and Social Sciences at Osaka University of Economics (Approval number: 2018-M01). Written informed consent from the participants’ legal guardian/next of kin was not required to participate in this study in accordance with the national legislation and the institutional requirements. Written informed consent was obtained from the individual(s) for the publication of any identifiable images or data included in this article.

## Author contributions

The research idea of this study came from the meeting of KF, KN, and ME. KF carried out data collection and statistical testing. KN performed synchrony analysis and revised the article. KF and ME drafted the article. All authors contributed to the article and approved the submitted version.

## Funding

This study was supported by the Research Grant for Collaborative Research (PI: KN) from the Osaka University of Economics. The funder had no role in the study design, data collection and analysis, decision to publish, or manuscript preparation.

## Conflict of interest

The authors declare that the research was conducted in the absence of any commercial or financial relationships that could be construed as a potential conflict of interest.

## Publisher’s note

All claims expressed in this article are solely those of the authors and do not necessarily represent those of their affiliated organizations, or those of the publisher, the editors and the reviewers. Any product that may be evaluated in this article, or claim that may be made by its manufacturer, is not guaranteed or endorsed by the publisher.
