# Supplementary material for: Antiphase synchrony increases perceived entitativity and uniqueness: A joint hand-clapping task
Source: Front Psychol. 2023 Mar 13;14:1069660. doi: 10.3389/fpsyg.2023.1069660 (PMC10040872; doi:10.3389/fpsyg.2023.1069660)
Supplement: Supplementary file 1 [file Data_Sheet_1.PDF]

Anti-phase synchrony increases perceived entitativity and uniqueness: A joint hand-clapping task

1 SUPPLEMENTARY RESULTS

To see the pattern of when participants voluntarily clapped their hands and the effect of this on the self-report measure, we had the additional third session. The results of one-way ANOVA with a between factor (synchrony: anti-phase, in-phase, asynchrony) showed there was no significant difference for all the variables [$F\text{s}(2, 62) < 2.83$, $p\text{s} < .067$, $\eta^2 < .085$]. Thus, the overall average and correlation in the additional session was indicated in Table S1. The mean values of the phase synchrony suggested their movement was relatively closed to anti-phase synchrony (high Intercept coefficient) but unstable (low PLV) and showed unclear pattern (high RMS), which was similar to asynchrony condition in the second session. Self-report measures, therefore, showed a tendency to be close to asynchrony condition; low entitativity and IOS, and high uniqueness.

As for correlations, uniqueness and phase synchrony variables were significantly correlated; the PLV, the stability of synchrony across the session, and Intercept coefficient, the overall relative phase tendency, indicated the negative association, whereas the RMS, the variance of the relative phase between the confederate and the participant, was positively associated with uniqueness. The RMS also showed significant association to IOS.

		M	*SD*	1	2	3	4	5
1	PLV	0.801	0.166	-				
2	Intercept coefficient	0.686	0.299	.059	-			
3	RMS	0.645	0.168	$-.648^{**}$	$-.029$	-		
4	Entitativity	2.350	1.317	.195	$-.045$	$-.200$	-	
5	Uniqueness	4.835	1.332	$-.269^{*}$	$-.271^{*}$	$.324^{**}$	$-.213$	-
6	IOS	2.446	1.659	.237	$-.135$	$-.291^{*}$	$.595^{**}$	$-.154$

Table S1. Mean, SD, and correlation matrix of phase synchrony and self-report measures in the third session. PLV: Phase Locking Value. RMS: Root Mean Square. IOS: the Inclusion of the Other in Self scale

2 SUPPLEMENTARY DISCUSSION

The main findings of this study were obtained under the unilateral synchronization, especially, the situation of *perceived* synchrony where the participant moves periodically and the other (i.e., confederate) adjusted his or her movement, which provided the participant more passive experience of synchrony. In the main session of this study, the second session, the participants clapped their hands with the beat of the metronome, and the confederate “adjusted” their movement according to the experimental assignment, which means the participant was literally a leader and the confederate was a follower. A leader in synchronous movement feels self-identity derived from the sense of control.

In contrast, the experience of actively adjusting one’s movements to achieve synchrony can be described as *produced* synchrony, which corresponds to the third session in this study. The results of the third session showed that the participants did not move synchronously to the partner and there was no systematic difference according to the second session. The obtained patterns do not support the previous studies that

suggests in- and anti-phase synchrony is a stable attractor. This might be because the participants were bored to the synchronous movement then required some changes on purpose. While stable synchrony was not seen, the results supported previous studies; less stable and variant asynchrony movement increased the self-identity and reduced self-other overlap.
